# Successful treatment of ciliary body medulloepithelioma with intraocular melphalan chemotherapy: a case report

**DOI:** 10.1186/s12886-020-01512-y

**Published:** 2020-06-18

**Authors:** Christina Stathopoulos, Marie-Claire Gaillard, Julie Schneider, Francis L. Munier

**Affiliations:** Department of Ophthalmology, Jules-Gonin Eye Hospital, Fondation Asile des Aveugles, University of Lausanne, Avenue de France 15, 1000 Lausanne 7, Vaud Switzerland

**Keywords:** Intraocular medulloepithelioma, Melphalan, Intracameral injection, Intravitreal injection

## Abstract

**Background:**

Intraocular medulloepithelioma is commonly treated with primary enucleation. Conservative treatment options include brachytherapy, local resection and/or cryotherapy in selected cases. We report for the first time the use of targeted chemotherapy to treat a ciliary body medulloepithelioma with aqueous and vitreous seeding.

**Case presentation:**

A 17-month-old boy with a diagnosis of ciliary body medulloepithelioma with concomitant seeding and neovascular glaucoma in the right eye was seen for a second opinion after parental refusal of enucleation. Examination under anesthesia showed multiple free-floating cysts in the pupillary area associated with iris neovascularization and a subluxated and notched lens. Ultrasound biomicroscopy revealed a partially cystic mass adjacent to the ciliary body between the 5 and 9 o’clock meridians as well as multiple nodules in the posterior chamber invading the anterior vitreous inferiorly. Fluorescein angiography demonstrated peripheral retinal ischemia. Left eye was unremarkable. Diagnosis of intraocular medulloepithelioma with no extraocular invasion was confirmed and conservative treatment initiated with combined intracameral and intravitreal melphalan injections given according to the previously described safety-enhanced technique. Ciliary tumor and seeding totally regressed after a total of 3 combined intracameral (total dose 8.1 μg) and intravitreal (total dose 70 μg) melphalan injections given every 7–10 days. Ischemic retina was treated with cryoablation as necessary. Three years later, *ab interno* trabeculotomy followed by 360° gonioscopy-assisted transluminal trabeculotomy 6 months later was performed for uncontrolled intraocular pressure despite antihypertensive drugs combined to cyclophotocoagulation and 7 intravitreal anti-VEGF injections for recurrent iris neovascularization. Cataract was removed at the same operative time. The child has remained disease- and metastasis-free at a 5-year follow-up since the last melphalan injection (25-month follow-up after the combined lensectomy-trabeculotomy) with a controlled intraocular pressure under topical quadritherapy and a best corrected Snellen visual acuity of 0.08.

**Conclusions:**

We report for the first time complete regression of a non-infiltrating ciliary body medulloepithelioma with seeding achieved with only a small number of intracameral and intravitreal melphalan injections. Concomitant secondary neovascular glaucoma and cataract needed appropriate management to allow long-term eye and vision preservation.

## Background

Intraocular medulloepithelioma is a rare, nonhereditary, neuroepithelial tumor, usually arising from the nonpigmented ciliary epithelium of the ciliary body, more rarely the optic nerve, the retina or the iris [1]. Its exact incidence is not known. Almost all cases are unilateral, with no gender or racial predilection. Diagnosis is made within the first decade of life in about 80% of the cases [2]. Most patients present with loss of vision, pain, leukocoria or a mass appearing in the anterior chamber [1, 3]. In 5% of the cases, a systemic association with pleuropulmonary blastoma caused by heterozygous *DICER1* mutations has been found [1]. Histopathologically, intraocular medulloepithelioma can be classified into nonteratoid and teratoid types and both can be benign or malignant. Even in the malignant form, these slowly growing tumors do not tend, however, to metastatize, unless extraocular extension occurs [1–3].

Characteristic features of ciliary body medulloepithelioma include a grey-white to fleshy pink mass with a various quantity of cysts adjacent to the ciliary body, a lens notching with or without subluxation and a neoplastic cyclitic membrane [3, 4]. The cysts within the mass may break away and float freely into the aqueous or the vitreous cavity. Coexistence of persistent hyperplastic primary vitreous has been reported in 20% of the cases [3, 4]. Around 50% of the cases are complicated with glaucoma and/or cataract [1–3]. Treatment options include enucleation [1–4], plaque brachytherapy [1, 2, 5], surgical resection [2, 4, 6] and cryotherapy [2, 4].

Here we report for the first time a case of intraocular medulloepithelioma with seeding at presentation successfully treated with intracameral and intravitreal melphalan chemotherapy.

## Case presentation

**Fig. 1:**
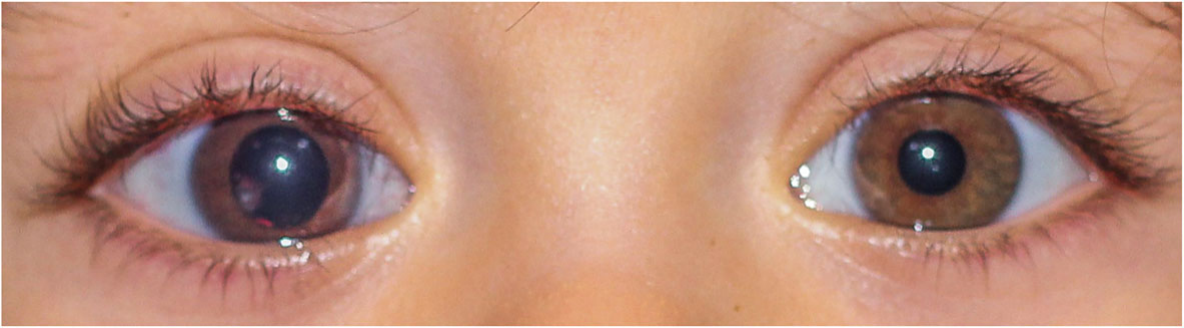
Mydriasis with sectorial inferotemporal leukocoria in the right eye of the 17-month-old child as presenting sign of intraocular medulloepithelioma. Note the darker right eye appearance due to iris neovascularization and the inferior transillumination due to the subluxated colobomatous lens

**Fig. 2:**
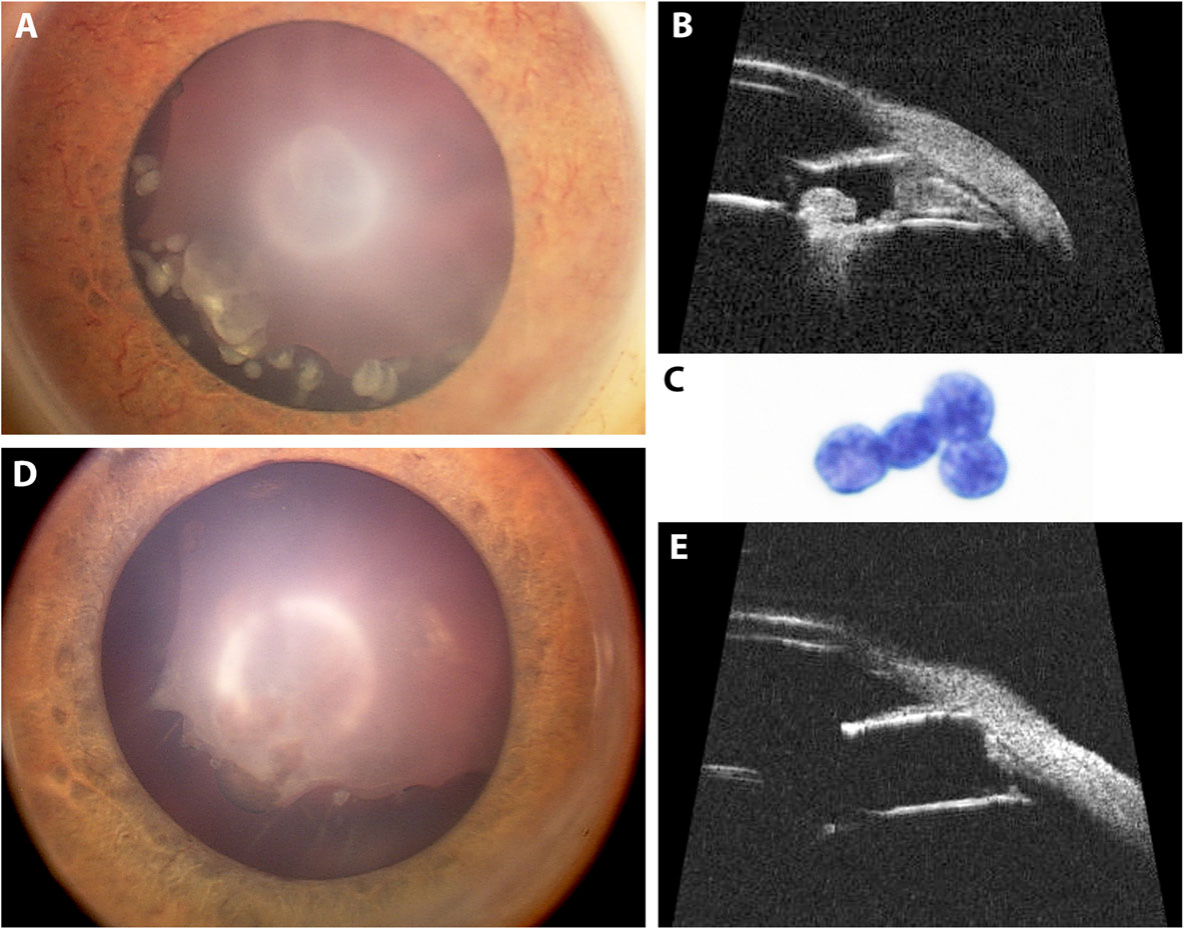
**a*.*** Anterior segment chamber photography of the right eye at diagnosis showing iris neovascularization, multiple free-floating cysts in the pupillary area as well as a subluxated and notched lens. **b.** Ultrasound biomicroscopy (35 MHz) on the 6 o’clock meridian displaying an iridocorneal angle closure and an irregular-surfaced mass with intratumoral cysts adjacent to the ciliary body, growing from the ciliary epithelium into the posterior chamber without invading the ciliary muscle. Clumps of hyperechogenic material are found in the posterior chamber (cysts) and the anterior vitreous (dust). **c.** Cytology analysis of the aqueous tap performed before treatment showing aggregates of malignant small cells with high nuclear/cytoplasmic ratio and finely granular chromatin. **d-e.** Anterior segment photography and ultrasound biomicroscopy one month after 3 intracameral and 3 intravitreal melphalan injections showing complete response of the primary tumor and the aqueous/vitreous seeding. Iris neovascularization had transiently regressed after concomitant intravitreal anti-vascular endothelial factor and cryoablation of the peripheral ischemic retina

A previously healthy 17-month-old boy was given topical antibiotics for a right eye redness appearing in the context of an ear, nose and throat infection. One week later, fixed mydriasis of the same eye was observed, and the child seen elsewhere for evaluation (Fig. 1). Ophthalmologic examination revealed an intraocular mass and the child was referred to a specialized oncology centre for further investigations. Diagnosis of intraocular medulloepithelioma with anterior chamber seeding and neovascular glaucoma was made and immediate enucleation advised. Parents refused the intervention and consulted us for a second opinion. Examination under general anesthesia showed multiple free-floating cysts in the pupillary area of the right eye with iris neovascularization and a notched lens that was subluxated superonasally (Fig. 2a). Intraocular pressure was controlled (17 mmHg) under topical beta-blocker and oral acetazolamide. Fundus examination was normal. Fluorescein angiography revealed anterior segment leakage with inferior peripheral retinal ischemia. Ultrasound biomicroscopy performed on the 12 meridians showed a complete iridocorneal angle closure and an irregular-surfaced, partially cystic mass adjacent to the ciliary body between the 5 and 9 o’clock meridians. Multiple nodules were invading the posterior chamber and anterior vitreous invasion was also seen inferiorly (Fig. 2b). Left eye examination was unremarkable. Clinical appearance and ultrasonographic features confirmed the diagnosis of ciliary body medulloepithelioma. There being no signs of extraocular extension nor ciliary infiltration, conservative treatment was initiated. After obtaining parents’ informed consent, combined intracameral (2.25 μg/0.15 ml) and intravitreal melphalan (30 μg/0.15 ml) injections given according to a previously described safety-enhanced technique to avoid potential extraocular tumor spread [7, 8]. Injected doses were similar to the ones used in the treatment of aqueous and vitreous seeding in retinoblastoma. Cytopathological analysis of the aqueous tap performed prior to injection showed small aggregates of malignant cells with high nuclear cytoplasmic ratio that were negative for synaptophysin on immunocytochemistry (Fig. 2c). Partial regression of both ciliary mass and seeding was observed 1 week later, encouraging treatment to be repeated. In total, 3 combined intracameral (total dose 8.1 μg) and intravitreal (total dose 70 μg) melphalan injections were given every 7–10 days, resulting in complete response of the main tumor (documented on UBM after 2 combined injections) and aqueous/vitreous seeding (observed after 3 combined injections) (Fig. 2d-e). One week after the final injections, dense anterior chamber hyphema related to persistent iris neovascularization occurred. After careful anterior chamber washout under melphalan perfusion (15 μg/ml), iris neovascularization was managed with cryoablation of the peripheral retinal ischemia and intravitreal anti-vascular endothelial growth factor (anti-VEGF). Three years later, the patient underwent *ab interno* trabeculotomy followed by a 360° gonioscopy-assisted transluminal trabeculotomy 6 months later to manage uncontrolled intraocular pressure despite antihypertensive drugs combined with cyclophotocoagulation and 7 intravitreal anti-VEGF injections for recurrent iris neovascularization. Cataract surgery with posterior capsulorhexis, anterior vitrectomy and sulcus implantation of a three-piece intraocular lens was performed during the first glaucoma surgery. Cytopathologic analysis of the vitrectomy fluid was negative for tumor cells. One year after the cataract was removed, YAG-laser capsulotomy was performed for posterior capsular lens opacification. The child has remained disease and metastasis-free at a 5-year follow-up since the last chemotherapy with a controlled intraocular pressure under topical quadritherapy and a best-corrected Snellen visual acuity of 0.08.

## Discussion and conclusions

Intraocular medulloepithelioma is a rare tumor of unknown incidence but still represents the most frequent pediatric primary malignant intraocular tumor after retinoblastoma [9]. Standards of care do not exist. Large size or invasive medulloepitheliomas are commonly treated with primary enucleation [3, 4, 10, 11]. Exenteration in combination with irradiation and/or adjuvant chemotherapy may be necessary in cases with orbital involvement [3, 12]. Small or medium-size well-circumscribed tumors can be treated conservatively with I-125 or Ru-106 plaque radiotherapy [1, 2, 5]. Local resection with iridocyclectomy or partial lamellar sclerouvectomy are usually insufficient, displaying a high recurrence rate requiring secondary enucleation [4, 6, 13]. To date, the role of first line chemotherapy for conservative management of medulloepithelioma is not known, as its use has been restricted to adjuvant or neoadjuvant therapy of cases with orbital invasion and/or metastasis [14–17].

In this report, we describe for the first time the conservative management of a non-infiltrating ciliary body medulloepithelioma complicated with seeding at presentation using intraocular melphalan injections. Presence of seeding was so far considered as an indication for enucleation [4]. Melphalan is a well-known drug employed in the treatment of various solid and hematopoietic cancers such as ovarian cancer, breast cancer or multiple myeloma [18]. Its cytotoxic activity is mediated by its DNA alkylating properties, which leads to cell death [18]. Melphalan injections directly into the vitreous or the aqueous humor via intravitreal or intracameral injections respectively, allow high intraocular tumoricidal drug concentrations to be reached, while by-passing systemic adverse effects and are therefore currently the treatment of choice for vitreous and aqueous seeding in retinoblastoma [19, 20]. Recently intravitreal melphalan has also been used to salvage two cases with primary vitreoretinal lymphoma [21]. In our case, intraocular melphalan alone was sufficient to achieve complete regression, not only of the seeding but also of the primary solid tumor, presumably because of its friable nature and the absence of concomitant ciliary muscle infiltration. Injected doses were decided based on our experience with retinoblastoma. The minimal tumoricidal drug concentration for intraocular medulloepithelioma remains to be established. Posterior capsular cataract needing surgery 3 years after treatment completion was the only treatment-related adverse effect observed in our patient.

In conclusion, we report herein for the first time a case of non-invasive ciliary body medulloepithelioma with seeding at presentation successfully treated with only a small number of intraocular melphalan injections. To our knowledge, this is the first case of intraocular medulloepthelioma with seeding to be managed conservatively. Concomitant secondary neovascular glaucoma and cataract needed appropriate management to allow long-term eye and vision preservation. Further studies will help to better define the role of targeted chemotherapy for intraocular medulloepithelioma in the future.

## Data Availability

Not applicable.

## Abbreviations

UBM: Ultrasound biomicroscopy
VEGF: Vascular endothelial growth factor
YAG: Yttrium aluminium garnet
